# Regulation of alveolar macrophage death in acute lung inflammation

**DOI:** 10.1186/s12931-018-0756-5

**Published:** 2018-03-27

**Authors:** Erica K Y Fan, Jie Fan

**Affiliations:** 1 Kenneth P. Dietrich School of Arts & Sciences, University of Pittsburgh, Pittsburgh, PA 15260 USA; 2 Department of Surgery, University of Pittsburgh School of Medicine, Pittsburgh, PA 15213 USA; 3 Research and Development, Veterans Affairs Pittsburgh Healthcare System, Pittsburgh, PA 15240 USA; 4 McGowan Institute for Regenerative Medicine, University of Pittsburgh, Pittsburgh, PA 15219 USA

**Keywords:** Acute lung injury, Macrophages, Cell death, Pyroptosis, Necroptosis, Autophagy

## Abstract

Acute lung injury (ALI) and its severe form, known as acute respiratory distress syndrome (ARDS), are caused by direct pulmonary insults and indirect systemic inflammatory responses that result from conditions such as sepsis, trauma, and major surgery. The reciprocal influences between pulmonary and systemic inflammation augments the inflammatory process in the lung and promotes the development of ALI. Emerging evidence has revealed that alveolar macrophage (AM) death plays important roles in the progression of lung inflammation through its influence on other immune cell populations in the lung. Cell death and tissue inflammation form a positive feedback cycle, ultimately leading to exaggerated inflammation and development of disease. Pharmacological manipulation of AM death signals may serve as a logical therapeutic strategy for ALI/ARDS. This review will focus on recent advances in the regulation and underlying mechanisms of AM death as well as the influence of AM death on the development of ALI.

## Background

Regulated cell death is critical for the development and maintenance of an organism. In the 1970s, apoptosis was noted as “the only form of regulated cell death” [1–3]. As the field developed and progressed, a variety of regulated cell deaths were characterized, and depending on the signaling pathways involved, these different types of cell death resulted in either lytic or non-lytic morphology [4]. For example, apoptosis [3, 5] is a non-lytic and usually non-immunogenic form of cell death; whereas, necroptosis [6–8], pyroptosis [9], and NETosis [10] are lytic and highly inflammatory. In host defense, cell death can be used defensively, reducing infections by separating unaffected cells from infected cells; however, cell death can also increase inflammation.

The lung is one of the most important target organs for pro-inflammatory mediators secreted and released globally during sepsis [11] and trauma [12–14]. These severe pathologies are often followed by the development of acute lung injury (ALI) and acute respiratory distress syndrome (ARDS), which are characterized by pulmonary infiltrates, hypoxemia, and injury to both the vascular endothelium and lung alveolar epithelium. Alveolar macrophages (AM) account for approximately 95% of airspace leukocytes [15], and through the synthesis and release of various inflammatory mediators, AM critically influence the development of ALI following infection and non-infectious stimuli [16, 17]. It is now clear that AM and other immune cells work in concert in the regulation of lung inflammation [18]. For example, while sepsis and trauma can lead to ALI and ARDS, equally, pulmonary infection and diffuse injury can cause systemic inflammatory response syndrome (SIRS), sepsis, and even septic shock [19]. These clinical syndromes lead to significant morbidity and mortality in intensive care units [20] and underscore the interplay between pulmonary and systemic inflammation in promoting disease progression.

Emerging evidence has revealed that AM cell death plays important roles in influencing the progression of lung inflammation [21–23]. There is increasing recognition that inflammation and cell death reciprocally affect each other and form an auto-amplification loop of these two factors, which in turn exaggerates inflammation [24]. Therefore, pharmacological manipulation of AM death signals may potentially serve as a logical therapeutic strategy for ALI/ARDS. This review will focus on recent advances in the regulation of AM death and underlying mechanisms as well as the influence of AM death on the development of acute lung inflammation.

### Alveolar macrophage Pyroptosis

Pyroptosis is a form of regulated cell death that is both inflammatory and immunogenic. Cell pyroptosis protects multicellular organisms from invading pathogenic microbial infections; however, pyroptosis can cause local and systemic inflammation and even lead to lethal septic shock [25]. Pyroptosis is dependent on the activation of caspase-1 or caspase 11/4/5, which cleaves gasdermin D (GSDMD), a member of a family of conserved proteins that includes gasdermin A,B,C,D, E, and DFNB59 [26], most of which have been shown to have pore-forming activity. Cleavage of GSDMD leads to the separation of its N-terminal pore-forming domain (PFD) from the C-terminal repressor domain followed by PFD oligomerization and formation of large pores in the cell plasma membrane, causing cell swelling and membrane rupture. As such, pyroptosis is defined as a gasdermin-mediated regulated necrosis [25, 26].

The inflammasome, a protein complex that activates caspase-1 and the secretion of cytokines IL-1β and IL-18, is one of the machineries that promote pyroptosis. The inflammasome is composed of sensor molecules, i.e. Nod-like receptor (NLRP1, 3, 6, 7, 12, NLRC4), AIM2, or Pyrin, in addition to an adaptor molecule apoptosis-associated speck-like protein containing CARD (ASC) and procaspase-1 [27, 28]. Procaspase-1 associates with the ASC focus via CARD-CARD interactions, which leads to dimerization and proximity-induced proteolytic processing of procaspase-1 into two subunits, p10 and p20, thus activating caspase-1. Activated caspase-1 further cleaves pro-IL-1β, pro-IL-18, and GSDMD. Normally, inflammasome formation requires a canonical two-step mechanism. Taking the NLRP3 inflammasome as an example, the first signal (e.g. stimulation of TLR4 or another pattern recognition receptor) stimulates NF-kappa B and enhances the expression and synthesis of NLRP3; the second signal induces NLRP3 inflammasome assembly. Common second signals include binding of P2X purinoreceptor 7 (P2X7R) by adenosine triphosphate (ATP), K^+^ efflux, lysosome destabilization caused by urate crystals, and generation of DNA and reactive oxygen species (ROS) in the mitochondria [29, 30].

Franklin et al. have shown that ASC foci that collect in the extracellular space following pyroptosis can promote IL-1β maturation [31]. Macrophage phagocytosis of ASC foci provokes lysosome rupture, soluble ASC nucleation, and the cleavage of IL-1β in the recipient cells [31]. These observations suggest an important mechanism, in which inflammasomes released from pyroptotic cells act as danger signals to amplify macrophage inflammatory responses.

IL-1β is an important proinflammatory cytokine that stimulates leukocyte expression of various cytokines and chemokines and leukocyte migration into tissue and organs [32]. IL-18 induces IFNγ expression and secretion and is critical for the activation of macrophages, T-cells, and other immune cells [33]. It has been reported that cell lysis suppressed by pharmacological inhibitors did not prevent caspase-1-dependent pore formation on the plasma membrane and cytokine release, suggesting that release of IL-1β and IL-18 is not a cell lysis-dependent process, whereas, cell lysis and cytokine secretion are both consequent downstream events following caspase-1-dependent plasma membrane pore formation [34]. Caspase-1-dependent pyroptosis may not occur in all cell types. For example, caspase-1 activation in epithelial cells prevents cell pyroptosis through stimulating membrane repair function as a response to the membrane damage caused by pore-forming toxins aerolysin and α-toxin [35].

**Fig. 1:**
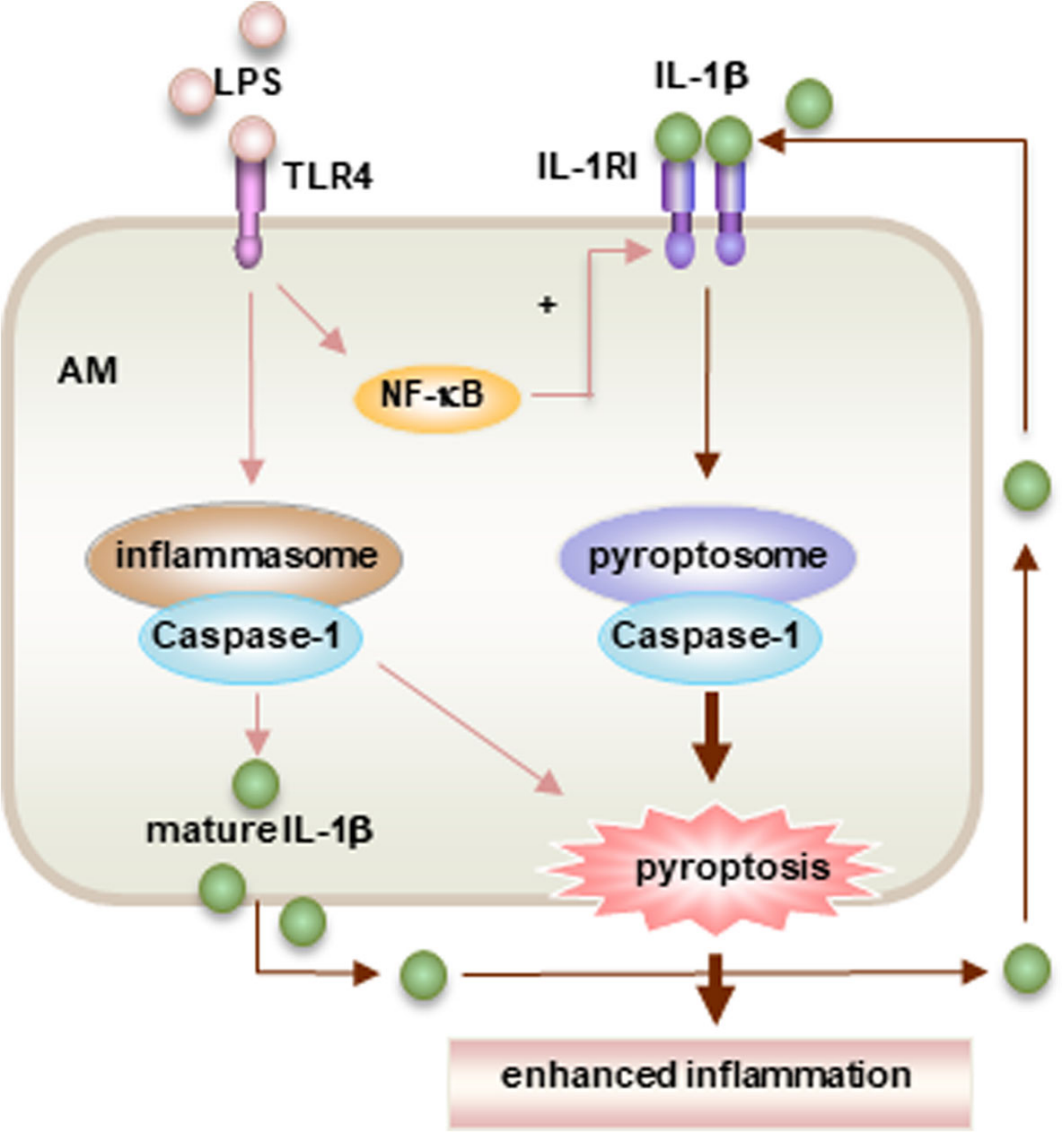
IL-1β-IL-1RI-mediateded AM pyroptosis and lung inflammation in response to LPS. In AM LPS-TLR4 signaling not only activates Nlrp3 inflammasome activation and subsequent release of IL-1β, but also up-regulates IL-1RI cell surface expression through MyD88 and NF-κB dependent signaling. The upregulated IL-1RI, therefore, sensitizes AM to IL-1β and results in pyroptosome activation, which in turn leads to AM pyroptosis, a type of caspase-1-dependent inflammatory cell death, and subsequent exaggerated lung inflammation (This figure is adapted from the Ref. [36]. Adapted with permission)

A recent study explored the role of LPS-activated IL-1β release and IL-1RI upregulation in the progression of lung injury in the context of AM pyroptosis [36]. The study showed that in AM, LPS acting through TLR4-MyD88-NF-ĸB dependent signaling stimulates Nlrp3 inflammasome activation and consequent secretion of IL-1β. Importantly, LPS upregulates IL-1RI surface expression on AM, which sensitizes AM to IL-1β and results in pyroptosome formation and subsequent AM pyroptosis. Furthermore, the study demonstrated that AM pyroptosis aggravates lung inflammatory responses (Fig. 1) [36]. The data reveals a new mechanism by which LPS-activated upregulation of IL-1RI signaling promotes AM pyroptosis and amplifies lung injury.

ALI often occurs in post-hemorrhagic shock (HS) conditions. HS, as a result of major trauma or surgery, primes for ALI by exaggerating innate immune cell activation. Studies have shown that HS induces IL-1β expression and secretion, which in turn promotes the development of SIRS [37–43]. Regarding how HS enhances IL-1β expression, activation, and release from AM, LPS, while activating Nlrp3 inflammasomes in AM, also induce pyrin expression, which in turn suppresses inflammasome activation [44]. Pyrin, a 781 amino-acid protein, is a vital regulator of the inflammasome [45]. The N-terminal of pyrin constitutes a pyrin domain (PYD), a member of the death effector-fold domain [46, 47]. The PYD found in pyrin is also present in the nucleotide-binding oligomerization domain-like receptor protein (NLRP; the nomenclature used in mouse is Nlrp). Importantly, LPS-mediated upregulation of IL-10 enhances pyrin expression, which, particularly in later phases of inflammation, serves as a potent negative feedback mechanism to regulate inflammasome activation. However, HS-mediated suppression of IL-10 expression in AM attenuates the upregulation of pyrin in AM and lung endothelial cells, and therefore significantly enhances inflammasome activation and IL-1β secretion in the lungs. This study demonstrates a mechanism by which HS suppresses negative feedback regulation of the Nlrp3 inflammasome to enhance IL-1β secretion in response to subsequent LPS challenge, and thus primes the host for inflammation [44].

HS also sensitizes AM responses to bacterial products through upregulating pattern recognition receptor (PPR) expression in AM [48, 49]. Previous studies have shown that HS-activated neutrophils plays an important role in AM priming, particularly, in mediating TLR4 and TLR2 crosstalk in AM [48]. The study demonstrated with a mouse model of HS followed by intratracheal injection (i.t.) of LPS that HS augments LPS upregulation of TLR2 expression in AM through activation of IL-1 receptor associated kinase 4 (IRAK 4) [49, 50]. However, in mice with neutrophils depletion, HS/LPS-upregulated TLR2 expression was markedly decreased, and this decrease was reversed upon replenishment with neutrophils collected from HS mice, but not from sham-operated mice. Furthermore, the study demonstrated that reactive oxygen species (ROS) derived from neutrophil NADPH oxidase mediates TLR4-TLR2 crosstalk in AM. The enhanced TLR2 upregulation in AM augmented the AM inflammatory response to sequential stimulations of LPS and peptidoglycan (PGN), a TLR2 ligand. These results indicate that TLR4-upregulated TLR2 expression in AM, which is enhanced by HS-activated neutrophils, serves as an imperative positive-feedback mechanism underlying HS-primed activation of AM [49, 50].

**Fig. 2:**
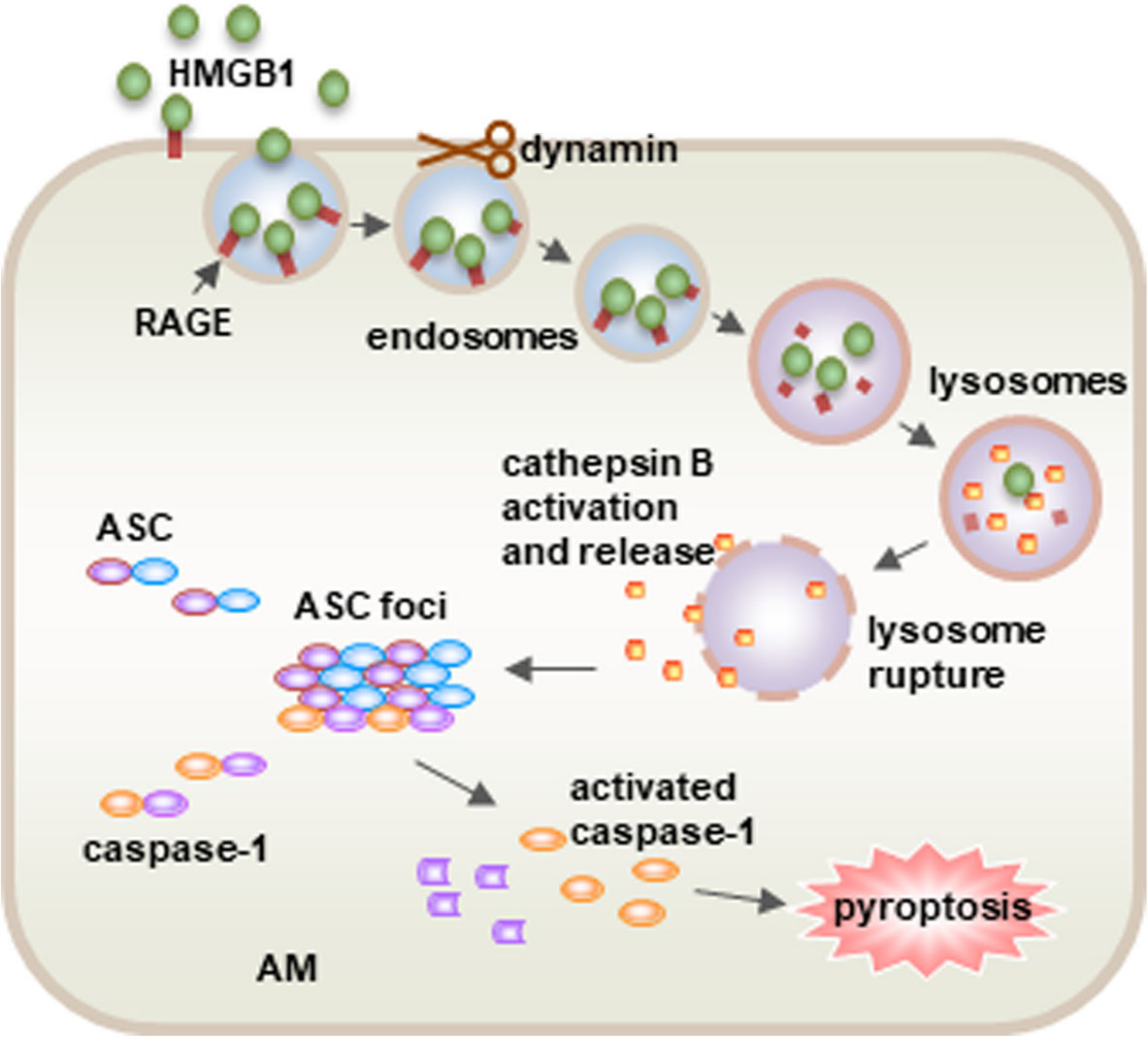
Endocytosis of HMGB1 induces pyroptosis. HMGB1 acting through RAGE on macrophages triggers dynamin-dependent endocytosis of HMGB1, which in turn initiates a cascade of cellular and molecular events. These include CatB activation and release from ruptured lysosome followed by pyroptosome formation and caspase-1 activation, which serves as a mechanism underlying the HMGB1-induced pyroptosis (This figure is adapted from the Ref. [21]. Adapted with permission)

Inflammasome activation can be bypassed in macrophage pyroptosis formation induced by high mobility group box 1 (HMGB1). HMGB1, a ubiquitous nuclear protein that exists in virtually all cell types, is a damage-associated molecular pattern (DAMP) molecule [51–53]. HMGB1 is released from cells following infection and tissue injury and is a crucial inflammatory mediator to induce a range of cellular responses, which include cell chemotaxis and release of pro-inflammatory cytokines [54, 55]. HMGB1 binding to its cell surface receptors, including TLR2, TLR4, TLR9 and the receptor for advanced glycation end products (RAGE) is necessary for initiating inflammatory responses [52, 53]. We have reported an inflammasome-independent mechanism by which HMGB1 induces AM pyroptosis [21]. We demonstrated that HMGB1 binds to RAGE on AM and elicits HMGB1 endocytosis, a dynamin-required event, followed by a cascade of molecular events, which include cathepsin B (CatB) activation and release from lysosomes, and ACS foci (also known as pyroptosome) formation and caspase-1 activation (Fig. 2). These findings were further confirmed in an endotoxemia mouse model, suggesting pathological implications for AM pyroptosis in the progression of inflammatory responses [21].

Infection and trauma through release of pathogen-associated molecular pattern molecules (PAMPs) and DAMPs, respectively, activate inflammasomes [56, 57], which in turn may initiate cell necroptosis. Cullen et al. demonstrated that inflammasome activation is associated with “conventional necrosis” and that the necrotic plasma membrane permeabilization (PMP) allows for the efflux of K^+^ ions required for NLRP3 activation, as well as for the unspecific release of IL-1β [58]. Martín-Sánchez and colleagues [59] also reported that bee venom antimicrobial toxin peptide melittin, which is known to elicit an inflammatory reaction via the NLRP3 inflammasome in response to bee venom, induces canonical NLRP3 inflammasome activation by PMP and a reduction in the intracellular K^+^ concentration. This NLRP3 inflammasome requires the adaptor protein ASC to activate caspase-1 and induce IL-1β release; however, melittin is unable to induce large ASC aggregates. Melittin-induced PMP and subsequent cell death was independent of caspase-1, showing that rapid cell lysis driven by melittin excludes pyroptosis execution by caspase-1. These studies suggest that inflammasome activation and subsequent caspase-1 activation are not necessarily to induce pyroptosis, but lead to cell necroptosis, although how the signal elicits necroptosis pathway remains unclear.

Studies have shown that AM pyroptosis augments lung inflammation in response to intratracheal administration of LPS. AM pyroptosis promotes neutrophil migration into the lungs and increases alveolar concentrations of cytokines IL-6, TNFα, and IL-1β, and worsen histological manifestations of lung injury [36]. Thus, targeting AM pyroptosis may represent a therapeutic strategy for controlling lung inflammation.

### Alveolar macrophage necrosis and necroptosis

Traditionally, necrosis was viewed as an accidental subroutine, mostly resulting from very harsh physicochemical stimuli including abrupt changes in temperature, osmotic pressure, or pH [60]. However, recent evidence has shown that necrosis can also occur in a regulated manner called necroptosis. Necroptosis is regulated by a receptor-interacting protein kinase (RIPK)1-RIPK3 complex dependent pathway, and it is negatively regulated by Necrostatin-1 (Nec-1) [61]. Since AM necrosis initiated in inflammation is usually in a regulated form, we will use the term of AM necroptosis in this section. The class of death receptors that includes tumor necrosis factor receptor (TNFR)1, TNFR2, and Fas can induce necroptosis. Binding of TNF-α to TNFR1 causes dissociation of silencer of death domains (SODD) from the intracellular domain of TNFR1 [62], followed by complex I formation. Complex I is composed of TNFR1 and TNFR2, TNFR-associated death domain, Fas-associated death domain (FADD), RIPK1, E3 ubiquitin ligases, and inhibitor of apoptosis proteins (IAPs) cIAP1 and cIAP2 [63]. The downstream effects of RIPK1 depend on its ubiquitination state, thus positioning complex I at the crossroads of cell death and survival. Deubiquitination of RIPK1 suppresses the NF-κB pathway, which leads to cell death. TNFR1 activation with no integration of c-IAPs (e.g. with the treatment of IAP antagonists), or with inhibition of translation, or with deubiquitination of RIPK1 by the deubiquitinating enzyme CYLD may induce RIPK1 translocation to a secondary complex in the cytoplasm called complex II [64–66]. Complex II is composed of caspase-8, the death domain containing protein FADD, and cellular FADD-like IL-1β-converting enzyme-inhibitory protein. Activation of caspase-8 induces cleavage of RIPK1 and RIPK3 and promotes complex II into a pro-apoptosis state. Conversely, inhibition of the apoptosis pathway drives the formation of necrosomes, which are mainly composed of RIPK1 and RIPK3 and functions to promote necroptosis [67].

Noteworthy, the above described necroptosis signaling was explored mostly in cancer cell lines and Jurkat lymphocyte cells other than macrophages. Whether the necroptosis signaling is also valid in macrophages, or more particularly in AM, remains to be determined.

The pseudokinase mixed lineage kinase domain-like (MLKL) protein is a RIPK3 substrate and is required for necroptosis [68, 69]. MLKL recruitment and phosphorylation is caused by RIP homotypic interaction motif (RHIM)-dependent oligomerization and intra-molecular RIPK3 auto-phosphorylation [70, 71], which leads to induction of cell necroptosis [72]. Additionally, studies have reported that MLKL oligomerization induced by RIPK3 and its subsequent translocation to the plasma membrane is associated with its cytotoxic effects [73–76]: MLKL can precipitate plasma membrane rupture by binding to phosphatidylinositol phosphates (PIPs) [73, 75], causing changes in sodium and calcium influx through ion channels and consequently causing changes in intracellular osmotic pressure [74, 76, 77].

The mechanisms determining if a cell dies by apoptosis or necroptosis remain poorly understood [78]. Several studies suggest that expression levels of RIPK3 and MLKL are associated with the tendency for cells to undergo necroptosis [78–84]. Some studies suggest that catalytically inactive RIPK3 trends to lead to RIPK1-dependent apoptosis, whereas catalytically active RIPK3 induces necroptosis [85]. On the other hand, caspase-8 is the most important molecule that prevents necroptosis. Studies show that necroptosis can be achieved in vivo by a genetic defect compromising FADD–caspase-8 signaling, thus inhibiting apoptosis [86–89]. More studies, particularly, in vivo studies, are required to understand the mechanisms that determine the susceptibility of macrophages to necroptosis or apoptosis.

Some reports have demonstrated that RIPK1 can inhibit apoptosis and necroptosis through kinase-independent functions, which are important for late embryonic development and the prevention of inflammation in epithelial barriers [82, 90–93]. Lin and colleagues showed that RIPK1 prevents skin inflammation by inhibiting activation of RIPK3–MLKL-dependent necroptosis mediated by Z-DNA binding protein 1 (ZBP1, also known as DAI or DLM1) [94]. The Lin group showed that ZBP1 deficiency inhibited keratinocyte necroptosis and skin inflammation in mice with epidermis specific RIPK1 knockout. Mechanistically, ZBP1 interacted strongly with phosphorylated RIPK3 in cells expressing the conserved RHIM of endogenous mouse RIPK1 (RIPK1 RHIM), suggesting that the RIPK1 RHIM prevents ZBP1 from binding and activating RIPK3. Newton et al. reported that RIPK1 RHIM is also critical for preventing ZBP1/RIPK3/MLKL-dependent necroptosis during development [95]. However, future studies will need to address if the RIPK1 RHIM also plays an important role in the regulation of AM necroptosis in ALI.

**Fig. 3:**
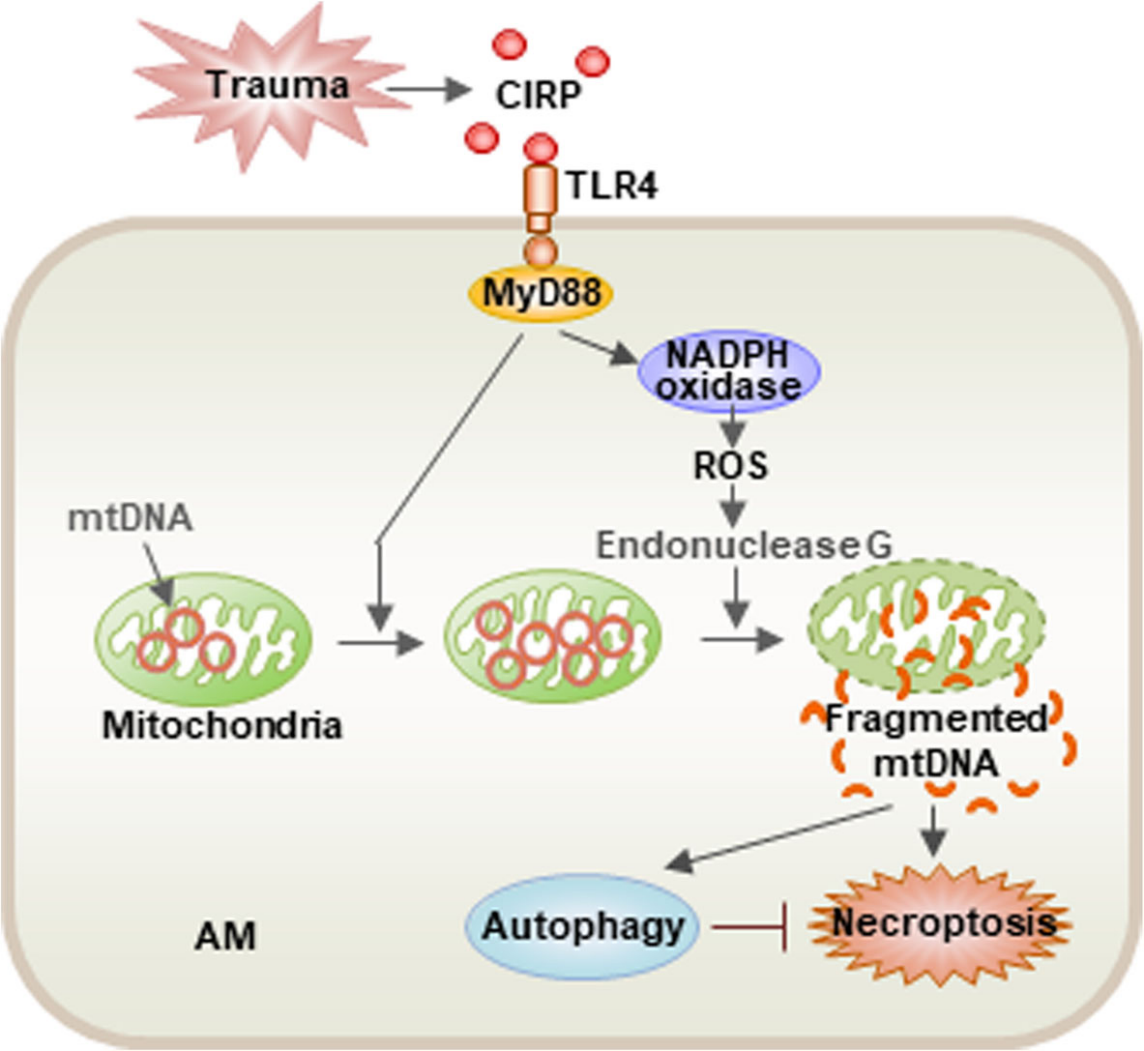
Trauma-induced mtDNA fragmentation regulates macrophage death. Trauma through CIRP-TLR4-MyD88 signaling induces NADPH oxidase activation and release of ROS, which activate endonuclease G. Endonuclease G directly fragments mtDNA, which triggers macrophage autophagy, as well as necroptosis by separate pathways. However, autophagy also suppresses macrophage necroptosis to limit local inflammation (This figure is adapted from the Ref. [96]. Adapted with permission)

Severe trauma often causes organ failure including ALI, in which AM play a critical role in the development of inflammation. Our group has recently reported a cold-inducible RNA-binding protein (CIRP)-mediated mechanism, by which trauma regulates AM death [96]. CIRP belongs to the cold shock protein family [97], and contains 172 amino acid residues (95% identical between human and mouse CIRP) that form a consensus sequence of N-terminal RNA-binding domain and C-terminal glycine-rich domain of nuclear proteins, which serve as RNA chaperones facilitating RNA translation [98, 99]. Studies have shown that CIRP is a DAMP molecule expressed during shock and trauma [100, 101], and it provokes a range of cellular inflammatory responses from inflammatory cytokines release to endothelial cell dysfunction [23]. We found that CRIP released from damaged tissue acts through TLR4 and MyD88-dependent signaling to induce mitochondrial DNA (mtDNA) fragmentation in AM [96]. This is achieved by ROS (particularly those originated from NADPH oxidase), which serve as key mediators of endonuclease G activation, which, in turn, directly regulates mtDNA fragmentation. Fragmented mtDNA then prompts AM autophagy and necroptosis via separate signaling pathways. Interestingly, autophagy suppresses AM necroptosis, thereby attenuating the propagation of local inflammation (Fig. 3) [96]. This study demonstrates an intracellular regulation of macrophage homeostasis in response to trauma.

**Fig. 4:**
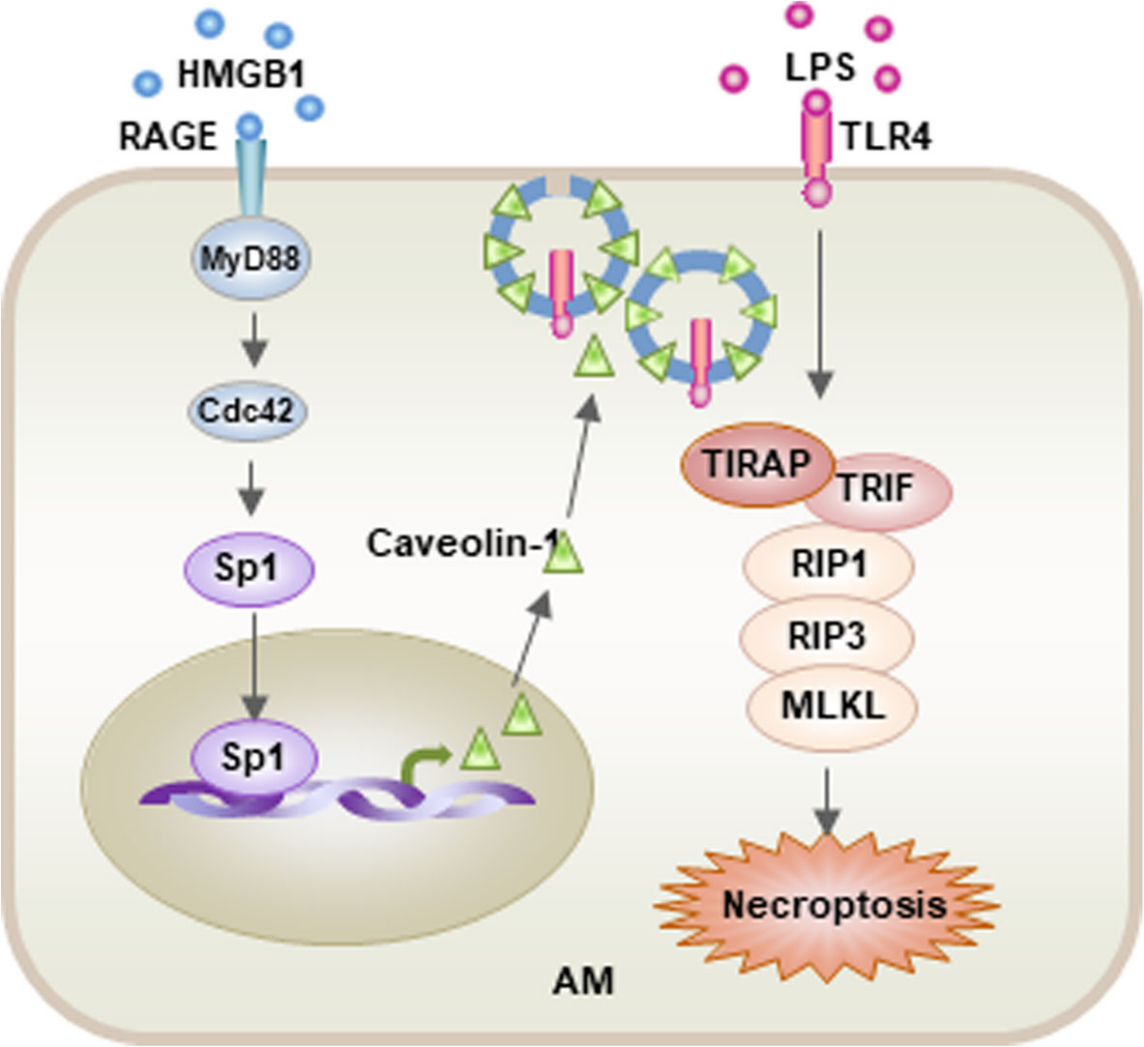
Model of the mechanism underlying tissue damage regulation of LPS-induced macrophage necroptosis. LPS acting through TLR4 promotes macrophage necroptosis. However, damaged tissue through HMGB1/RAGE signaling upregulates caveolin-1 expression in macrophage, which, in turn, induces caveolae-mediated TLR4 internalization and desensitization, thereby, ameliorates LPS-TLR4-induced macrophage necroptosis. RAGE-MyD88 signal activation of Cdc42 and the consequent nuclear translocation of Sp1 serve the mechanism of upregulation of caveolin-1 (This figure is adapted from the Ref. [22]. Adapted with permission)

The regulation of AM necroptosis is more complicated than previously thought, especially when multiple conditions, i.e. trauma and infection, exist simultaneously. Tissue damage resulting from severe trauma and surgery often results in either infection or heightened susceptibility to infection. The reciprocal influences between tissue damage and infection are considered to be a determinant in regulating the host’s inflammatory responses. The role of trauma and tissue damage in the regulation of LPS-induced AM necroptosis and the underlying mechanism has been examined [22]. The studies demonstrated that LPS-TLR4 signaling promotes AM necroptosis; however, necroptosis is ameliorated by the release of HMGB1 from damaged tissue. The study further showed that HMGB1 binds to its receptor RAGE to promote upregulation of caveolin-1 expression, which induces caveolae-dependent TLR4 internalization and desensitization, and ultimately decreases AM necroptosis. Furthermore, the study illustrated that the activation of transcription factor Sp1 by the RAGE-MyD88-Cdc42 signaling pathway is a mechanism that mediates caveolin-1 transcriptional upregulation (Fig. 4) [22].

**Fig. 5:**
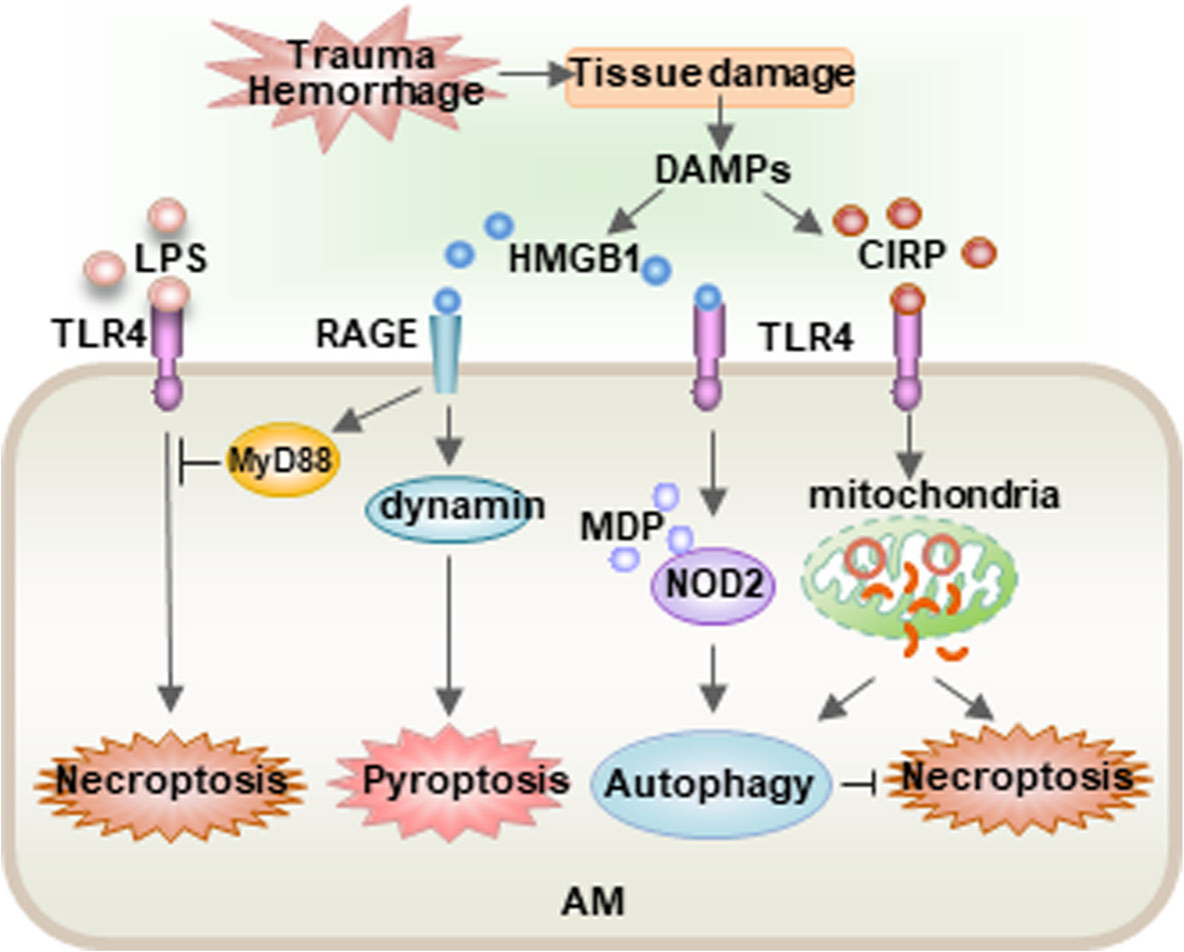
The complexity of AM death Regulation following infection, trauma, and HS. LPS, an important PAMP molecule derived from Gram negative bacteria acting through TLR4 promotes AM necroptosis. However, DAMP molecules released from tissue damage, which is a resultant of severe trauma and HS, significantly influence the regulation of AM death. For example, DAMP molecule HMGB1 acting through cell surface RAGE and MyD88-dependent pathway (as illustrated in Fig. 3) ameliorates the TLR4-mediated AM necroptosis. On the other aspect, HMGB1triggers RAGE-dynamin-dependent endocytosis of HMGB1, which promotes AM pyroptosis. HMGB1/TLR4 signaling upregulates NOD2 expression in AM and sensitizes them to subsequent NOD2 ligand MDP to induces autophagy in AM, which negatively regulates lung inflammation through feedback suppression of NOD2-RIP2 signaling and inflammasome activation. DAMP molecule CIRP acts through TLR4-MyD88 signaling to induce mtDNA fragmentation in AM, via a pathway in which NADPH oxidase-derived ROS served as a major mediator for the induction of endonuclease G, which, in turn, directly mediates mtDNA fragmentation. Fragmented mtDNA then triggered AM autophagy and necroptosis through separate signaling pathways, although autophagy also suppressed AM necroptosis, to attenuate propagation of local inflammation. Therefore, AM autophagy represents an intracellular negative regulation of AM death, whereas, pyroptosis serves as a dominant AM death form in a condition combined with infection, trauma and hemorrhage

It has been reported that a precedent trauma often primes for augmented inflammatory responses to infection and bacterial products through the release of DAMPs [37, 44, 48, 102, 103]. As described above, studies have shown that tissue damage-derived CIRP alone is able to induce AM necroptosis through mtDNA fragmentation [96]. Those results suggested that inhibition of DAMP signaling may diminish inflammatory responses to a secondary infection. However, the finding that tissue damage negatively regulates LPS-induced AM necroptosis through DAMP signaling unveils a feedback mechanism that attenuates the inflammatory response to a secondary LPS challenge. These results expose a previous unidentified protective role of DAMP molecules in limiting PAMP molecules-induced inflammation [22].

Studies have shown the effects of necroptosis on the development of systemic inflammation. In animal model of septic shock, RIPK3 deficiency prevents the remarkable increase in circulating cell death markers and promotes survival [104], suggesting that targeting RIPK3 in sepsis might be beneficial. Interestingly, caecal ligation puncture-mediated lethality was not restored in *Mlkl*-knockout mice [105], suggesting that the functions of RIPK3 and MLKL may not completely overlap in this context [106]. In a model of TNF-induced SIRS, pretreatment with necrostatin-1 or Nec-1 s strongly inhibited lethality [107], and this finding was supported by studies showing that RIPK1 kinase-inactive mice are also resistant to the lethality of TNF-induced SIRS and to the lethality of septic shock induced by a combination of TNF and Z-VAD-FMK [106, 108, 109]. Together, these data suggest that RIPK1 and RIPK3 inhibitors might be effective in treating SIRS and related diseases by targeting not only necroptosis but also other pro-inflammatory conditions regulated by these kinases [106].

### Alveolar macrophage autophagy

Autophagy is an evolutionarily conserved and genetically regulated pathway that serves to degrade and clear subcellular components [110, 111]. Autophagy was previously classified as a caspase-independent necrosis-like cell death associated with intracellular autophagosomes accumulation [112]. However, the causal relationship between autophagy and cell death has yet been to be defined, and thus, this classification remains controversial [113, 114].

The dynamic autophagic process is mainly implemented by a series of proteins, encoded by the autophagy related genes (ATGs). ATG proteins work in concert to dynamically promote formation of pre-autophagosome, maturation of autophagosome, and fusion with lysosome. The core machinery of autophagy, consisting of the ATGs, is regulated by many signaling pathways such as the mechanistic target of rapamycin (mTOR) pathway, the insulin pathway, and the ER stress response [115] . The signals that induce and regulate autophagy activation are still not fully elucidated. Since numerous review articles about autophagy basics are available [115–117], we will only focus on recent findings on the regulation and functions of AM autophagy in this review.

Autophagy can be activated by PRR signaling induced by DAMPs and PAMPs. For example, PAMPs can act through TLRs to induce autophagy [118, 119], and NLRs can cooperate with ATGs to confine autophagy [120, 121]. Inflammatory cytokines, including IL-1 family members [122, 123] and IFNγ [124–126], also promote autophagy; conversely, T_H_2 cell-associated cytokines, i.e. IL-4 and IL-13, inhibit autophagy [125]. Many studies have shown that autophagy protects organisms from infection by degrading intracellular pathogens, i.e. bacteria, viruses, and protozoan pathogens, demonstrating an important role of autophagy in host defense [127–129].

Our study explored a previously unidentified function of neutrophils in counteracting the AM intracellular anti-inflammatory mechanism of autophagy [103]. In a HS mouse model, we demonstrated that sequential treatment of HS and intratracheal injection of muramyl dipeptide (MDP), a NOD2 ligand, activates HMGB1-TLR4 signaling in AM, which upregulates NOD2 expression and sensitizes the AM to MDP, and therefore amplifies lung inflammation [103]. Additionally, upregulated NOD2 signaling promotes autophagy formation in AM, which suppresses lung inflammation through the downregulation of NOD2-RIP2 signaling, as well as NLRP3 inflammasome activation. Importantly, the study also demonstrated that shock-activated neutrophils migrate into the alveolar space to counteract the anti-inflammatory effect of autophagy in AM through an NADPH oxidase-dependent pathway that induces IKKγ phosphorylation, NF-κB activation, and NLRP3 inflammasome activation, consequently augmenting post-HS lung inflammation. These findings suggest an important role for receptor cross-talk and cell-cell interaction in the complicated mechanisms of ALI [103].

A recent study revealed a suppressive role of autophagy in AM apoptosis in the early stages of hypoxia [130]. Geng group reported that, using a cell hypoxia-reoxygenation model and rat orthotopic left lung ischemia-reperfusion model, rapamycin decreases the unfolded protein response, which reduces endoplasmic reticulum stress-mediated apoptosis in the presence of oxygen deficiency. Rapamycin increases superoxide dismutase activities and decreases malondialdehyde levels, whereas 3-MA decreases superoxide dismutase activities and increases malondialdehyde levels. Thus, the investigators concluded that autophagy decreases AM apoptosis by attenuating ER stress and oxidative stress in the early stage of hypoxia in vitro and in vivo [130].

Lung a post-ischemia-reperfusion (I/R) remains one of the most common complications after various cardiopulmonary surgeries. Liu and colleagues reported that, in a minipig lung I/R injury model, left lung I/R injury triggered release of DAMPs, such as HMGB1 and heat shock protein 60 (HSP60), in the lung and production of inflammatory cytokine and activation of autophagy flux in AM. However, autophagy inhibition by knockdown of ATG7 or BECN1 markedly reduced DAMP-triggered production of inflammatory cytokines including IL-1β, TNF, and IL12 in AM, and these changes were a result of decreased activation of MAPK and NF-κB signaling. Furthermore, knockdown of ATG7 or BECN1 inhibited Lys63 (K63)-linked ubiquitination of TNF receptor-associated factor 6 (TRAF6) in DAMP-treated AM. These results indicate that autophagy triggered by DAMPs during lung I/R injury amplifies the inflammatory response through enhancing K63-linked ubiquitination of TRAF6 and activation of the downstream MAPK and NF-κB signaling [131].

Autophagy’s effect on the outcome of acute lung inflammation depends on the disease background, phase or stage of the progression of the lung inflammation, and the balance between inflammatory factors, including pro-inflammatory cell death and anti-inflammatory factors. Nonetheless, modulation of autophagy may serve as a potential therapy for ALI.

### Death signal interaction between alveolar macrophages and neutrophils

Emerging evidence has revealed that neutrophils play important roles in influencing the behavior and functions of other cell types in the progression of lung inflammation. For example, we have reported that neutrophil–derived oxidants mediate the interaction between neutrophils and lung endothelial cells (LEC) and upregulate TLR2 expression in LEC via TLR4 signaling. Upregulation of TLR2 results in the stable and augmented expression of ICAM-1 in response to LPS and peptidoglycan, which then subsequently promotes transendothelial neutrophils migration into the lungs [132]. The above-mentioned finding that neutrophils counteract autophagy-mediated anti-inflammatory mechanisms in AM is another instance of neutrophil behavioral control [103].

Indeed, neutrophils are an active cell population with diverse functions and characteristics, including migration, phagocytosis, degranulation, secretion of cytokines, and release of ROS and enzymes; thus, neutrophils are thought to play an active role in inducing functional changes in other cell populations. Recent observations by our group have shown that this is a bidirectional relationship: following HS, AM in the alveolar space can target neutrophils to induce cell death [133]. Using hemorrhagic shock animal models, the study explored a previously unidentified role of hemorrhagic shock-activated AM in inducing neutrophil necroptosis. The study showed that exosomes released from hemorrhagic shock-activated AM cause neutrophils to produce ROS, primarily derived from NADPH oxidase, and consequently promote neutrophil necroptosis [133].

Exosomes are cell-derived secreted vesicles with a bilipid membranous structure. Exosomes are 30–100 nm in size and contain RNA, proteins, and lipids [134]. Most cells can secrete exosomes under normal conditions; however, under pathologic or stressful conditions, exosome quantity or content may be altered [135]. Reports suggest that exosomes mediate intercellular communication, stimulate target cells, promote antigen presentation, transfer pathogens, and regulate immune responses [136]. We found that exosomes released from HS-activated AM induce mainly NADPH oxidase-derived ROS production inside neutrophils and subsequent promotion of necroptosis [133]. These findings therefore add a new role for exosomes in mediating cell-cell crosstalk in HS. This exosomes-mediated neutrophil death pathway may be a potential therapeutic strategy to target post-HS SIRS.

It has been predicted that the pro-inflammatory cell death form of necroptosis is involved in disease pathogenesis since animal studies provided strong evidence for this hypothesis [137]. The migration of human neutrophils to inflammatory sites was found to activate the RIPK3-MLKL pathway in tissue samples from patients with neutrophilic diseases including cutaneous vasculitis, ulcerative colitis, and psoriasis [138]. However, the role of neutrophil necroptosis in human pathologies, especially in ALI, remains to be further identified [78, 139]. Thus, the question of how does neutrophil necroptosis impact on lung inflammation is still open.

## Conclusion

The clinical and pathologic manifestations of ALI/ARDS are very similar, indicating the existence of common pathways that represent potential therapeutic targets. Fundamentally, ALI/ARDS reflect severe injury leading to dysfunction and compromise of the barrier properties of the pulmonary endothelium and epithelium as a consequence of an unregulated acute inflammatory response. We are still lacking effective pharmacotherapy that can increase the survival of ALI/ARDS patients. Studies have provided evidence for a connection between cell death and lung inflammation, and understanding of the impact of AM death on the progression of lung inflammation is critical in fully elucidating the mechanisms underlying ALI/ARDS. However, by reviewing the literature, we found that reports derived from translational and clinical research on the mechanism of cell death in ALI/ARDS are insufficient, and thus, the translational and clinical research is expected.

It is imperative to realize the therapeutic potential of targeting cell death pathways in ALI/ARDS patients. Following an initial event of inflammation, cell death and inflammation can induce each other and drive a local auto-amplification loop that leads to exaggerated cell death and inflammation [24]. Pharmacological inhibition of the autoamplification loop should ideally target the initial AM death, and thereby theoretically prevent the loop from developing. For instances, inhibitors could be used to prevent the AM necroptosis, such as Nec-1 [140, 141], Nec-1 s, Nec-33, and necrosulfonamide [142]; and to target pyroptosis, caspase inhibitors and inflammasome inhibitors, i.e. pralnacasan (VX-740; Vertex Pharmaceuticals) and belnacasan (VX-765; Vertex Pharmaceuticals) might be applied [143]. A combination therapy approach involving the use of such agents along with standard immunosuppression to further interfere with regulated cell death could also be considered [24].

Other forms of cell death that are also implicated in pathological settings but that are not discussed here include apoptosis, ferroptosis, parthanatos, NETosis, and CypD-mediated cell death. Apoptosis was long thought to be the only form of programmed cell death during homeostasis, development, and disease, and has been heavily studied and discussed in numerus review articles in literature. The gaps in our knowledge on cell death include the following: 1) whether different types of cell death signaling developed separately as responses to specific triggers or whether they represent parts of a signaling network that follow common regulatory mechanisms, such as energy devastation or ROS production; and 2) how these regulatory networks modulate shifts between different cell death routines [106]. As summarized in Fig. 5, the regulation of AM death is complex given the multitude of factors that simultaneously influence the pathway. Although we have accumulated knowledge on cell death, we are still a long way from a fully understanding of how these pathways converge or synergize in an ALI setting. Comprehensive understanding of the molecular mechanisms that regulate cell death will allow the development of strategies that control cell death, thereby developing novel interventions for ALI/ARDS.

## Abbreviations

ALI: Acute lung injury
AM: Alveolar macrophage
ARDS: Acute respiratory distress syndrome
ASC: Apoptosis-associated speck-like protein containing CARD
ATGs: Autophagy related genes
CatB: Cathepsin B
CIRP: Cold-inducible RNA-binding protein
DAMP: Damage-associated molecular pattern
FADD: Fas-associated death domain
GSDMD: Gasdermin D
P2X7R: P2X Purinoreceptor
HMGB1: High mobility group box 1
HS: Hemorrhagic shock
HSP60: Heat shock protein 60
IAPs: Inhibitor of apoptosis proteins
I/R: Ischemia-reperfusion
IRAK: 4 IL-1 receptor associated kinase 4
LEC: Lung endothelial cells
MDP: Muramyl dipeptide
MLKL: Mixed lineage kinase domain-like
mtDNA: Mitochondrial DNA
mTOR: Mechanistic target of rapamycin
NLRP: Nucleotide-binding oligomerization domain-like receptor protein
PAMP: Pathogen-associated molecular pattern molecules
PMP: Plasma membrane permeabilization
PFD: Pore-forming domain
PGN: Peptidoglycan
PIPs: Phosphatidylinositol phosphates
PPR: Pattern recognition receptor
PYD: Pyrin domain
RAGE: Receptor for advanced glycation end products
RCD: Regulated cell death
RIPK: Receptor-interacting protein kinase
ROS: Reactive oxygen species
SIRS: Systemic inflammatory response syndrome
SODD: Silencer of death domains
TLR: Toll-like receptor
TNFR: Tumor necrosis factor receptor
TRAF6: TNF receptor-associated factor 6
ZBP1: Z-DNA binding protein 1
